# Brain Structural Plasticity: From Adult Neurogenesis to Immature Neurons

**DOI:** 10.3389/fnins.2020.00075

**Published:** 2020-02-04

**Authors:** Chiara La Rosa, Roberta Parolisi, Luca Bonfanti

**Affiliations:** ^1^Neuroscience Institute Cavalieri Ottolenghi, Orbassano, Italy; ^2^Department of Veterinary Sciences, University of Turin, Turin, Italy

**Keywords:** neurogenesis, immature neurons, doublecortin, postnatal brain development, cerebral cortex

## Abstract

Brain structural plasticity is an extraordinary tool that allows the mature brain to adapt to environmental changes, to learn, to repair itself after lesions or disease, and to slow aging. A long history of neuroscience research led to fascinating discoveries of different types of plasticity, involving changes in the genetically determined structure of nervous tissue, up to the ultimate dream of neuronal replacement: a stem cell-driven “adult neurogenesis” (AN). Yet, this road does not seem a straight one, since mutable dogmas, conflicting results and conflicting interpretations continue to warm the field. As a result, after more than 10,000 papers published on AN, we still do not know its time course, rate or features with respect to other kinds of structural plasticity in our brain. The solution does not appear to be behind the next curve, as differences among mammals reveal a very complex landscape that cannot be easily understood from rodents models alone. By considering evolutionary aspects, some pitfalls in the interpretation of cell markers, and a novel population of undifferentiated cells that are not newly generated [immature neurons (INs)], we address some conflicting results and controversies in order to find the right road forward. We suggest that considering plasticity in a comparative framework might help assemble the evolutionary, anatomical and functional pieces of a very complex biological process with extraordinary translational potential.

## Graphical Abstract

Neurogenesis (present both in the embryonic and adult brain) is a multistep biological process spanning from the division of stem/progenitor cells to the functional integration of new neurons in neural circuits. “Immaturity” is a phase in this process, also occurring in cells that are generated before birth but retain molecular features of “youth” during adulthood. These immature neurons (INs) share markers with newly born neurons. All these cells express doublecortin (DCX), which therefore cannot be considered a unique marker for neurogenic processes. Present knowledge suggests that, despite the common cellular/molecular features shared among mammals, more complex processes, such as some forms of brain plasticity, may differ remarkably, with a general trend of reduced adult neurogenesis (AN) from rodents to large-brained species, and possible inverse tendency for INs.

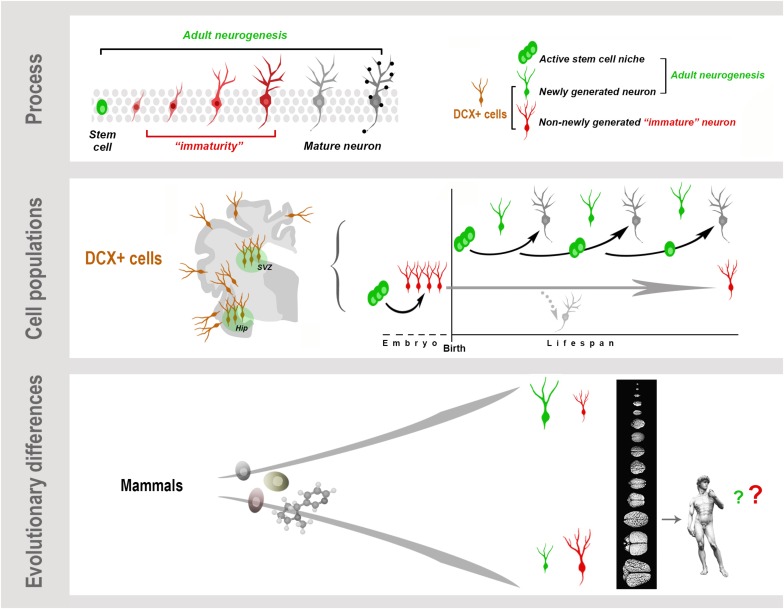

## Brief Historical Perspective: Revisiting A Never-Ending Story

Most neuronal plasticity in mammals relies on changes of synaptic contacts between pre-existing cells (synaptic strengthening, formation, elimination; Forrest et al., 2018). By considering the number of synapses in the brain (estimated in the trillions: 10^15^/mm^3^ in humans; Chklovskii et al., 2004), this can be considered the main potential for structural modification in the mammalian central nervous system (CNS). Nevertheless, this kind of plasticity does not add or replace neurons. Unlike non-mammalian vertebrates, which show remarkable neuronal cell renewal in their CNS (Ganz and Brand, 2016), the mammalian brain is far less capable of forming new neurons (Rakic, 1985; Weil et al., 2008; Bonfanti, 2011). The exception is a process called “adult neurogenesis” (AN), conferred by active stem cell niches that produce new neurons throughout life in restricted regions of the paleocortex (olfactory bulb) and archicortex (hippocampus) (Kempermann et al., 2015; Lim and Alvarez-Buylla, 2016). Yet, after 60 years of intense research and more than 10,000 peer-reviewed publications, we still do not know if our brain maintains such capability (Duque and Spector, 2019; Petrik and Encinas, 2019; Snyder, 2019). Although we have learned a lot about neural stem cell (NSC) biology and the molecular/cellular mechanisms that sustain neurogenesis in rodents (Bond et al., 2015; Kempermann et al., 2015; Lim and Alvarez-Buylla, 2016), direct analysis of human brain has produced many conflicting results (discussed in Arellano et al., 2018; Kempermann et al., 2018; Paredes et al., 2018; Parolisi et al., 2018; Petrik and Encinas, 2019). Here, we try to address such controversy by highlighting some biases and questionable interpretations, recurrent in the field, and by introducing the new concept of “immature neurons” (INs).

The intense research following the “re-discovery” of AN in mammals (starting from the seminal work of Lois and Alvarez-Buylla (1994), but adding to the pioneering studies of Joseph Altman and Fernando Nottebohm) were carried out almost exclusively using mice and rats. These studies were aimed to exploit endogenous and exogenous sources of stem/progenitor cells for therapeutic purposes (Bao and Song, 2018); however, the reparative capacity of mammalian AN was not sufficient, even in rodents (Bonfanti and Peretto, 2011; Lois and Kelsch, 2014). Further studies began to reveal that the main significance of the newborn neurons is linked to physiological roles, related to learning and adaptation to a changing environment (Kempermann, 2019). What appeared interesting is the discovery that AN is highly modulated by the internal/external environment and, ultimately, by lifestyle (Vivar and van Praag, 2017; Kempermann, 2019), which opened the road to prevention of age-related problems. These results also began to highlight the importance of evolutionary aspects (and constraints) revealed by the remarkable differences that exist among mammals (Barker et al., 2011; Amrein, 2015; Feliciano et al., 2015). As stated by Faykoo-Martinez et al. (2017): “Species-specific adaptations in brain and behavior are paramount to survival and reproduction in diverse ecological niches and it is naive to think AN escaped these evolutionary pressures” (see also Amrein, 2015; Lipp and Bonfanti, 2016). Subsequently, several studies addressed the issue of AN in a wider range of species, including wild-living and large-brained mammals that displayed a varied repertoire of anatomical and behavioral features, quite different from those of mice (reviewed in Barker et al., 2011; Amrein, 2015; Lipp and Bonfanti, 2016; Paredes et al., 2016; Parolisi et al., 2018). Though still too fragmentary to support exhaustive conclusions about phylogeny (much less function), this landscape of heterogeneity directs us to re-evaluate, discuss and better contextualize the observations obtained in rodents, especially in the perspective of translation to humans (analyzed in Lipp and Bonfanti, 2016; Paredes et al., 2016; Parolisi et al., 2018; Duque and Spector, 2019; Snyder, 2019). Comparative approaches strongly indicate that there is a decrease in the remarkable plastic events that lead to whole cell changes (i.e., AN) with increasing brain size. In an evolutionary framework, the absence/reduction of neurogenesis should not be viewed as a limit, rather as a requirement linked to increased computational capabilities. Unfortunately, this same fact turns into a “necessary evil” when brain repair is needed: a requirement for stability and a high rate of cell renewal, apparently, cannot coexist (Rakic, 1985; Arellano et al., 2018). Why then do some reports claim the existence of AN in humans? Several scientists in the field warn of high profile papers published on human AN that were technically flawed, their interpretations going well beyond what the data could support; some have never been reproduced (these aspects are thoroughly reviewed in Oppenheim, 2018; Duque and Spector, 2019). Apart from the soundness of data, a strong species bias exists in the neurogenesis literature, due to an overestimation of the universality of laboratory rodents as animal models (Amrein, 2015; Lipp and Bonfanti, 2016; Bolker, 2017; Faykoo-Martinez et al., 2017; Oppenheim, 2019). There is also a common misunderstanding that the putative existence of AN in primates suggests or provides evolutionary proof that the same process exists in humans. In fact, the few existing reports are on non-human primates (common marmosets and macaca), endowed with smaller, less gyrencephalic brains and lower computational capacity, compared to apes (Roth and Dicke, 2005). Systematic, quantitative studies in apes (family *Hominidae*) are still lacking and most studies carried out in monkeys suggest that very low levels of hippocampal neurogenesis persist during adulthood. In *Callithrix jacchus*, proliferating doublecortin (DCX)+ neuroblasts were virtually absent in adults and markers of cell proliferation and immaturity declined with age (Amrein et al., 2015). In another study involving *Macaca mulatta* and *Macaca fascicularis*, the estimated rate of hippocampal neurogenesis was approximately 10 times lower than in adult rodents (Kornack and Rakic, 1999). These data, along with evidence that AN is virtually absent in cetaceans (Patzke et al., 2015; Parolisi et al., 2017), do provide strong support for declining rates of AN in large-brained mammals (Paredes et al., 2016).

The reasons for some of these misunderstandings are analyzed in the next paragraph.

## Neurogenic Processes: Well-Defined Origin, Ill-Defined Markers, Uneven Outcome

### Origin

**FIGURE 1:**
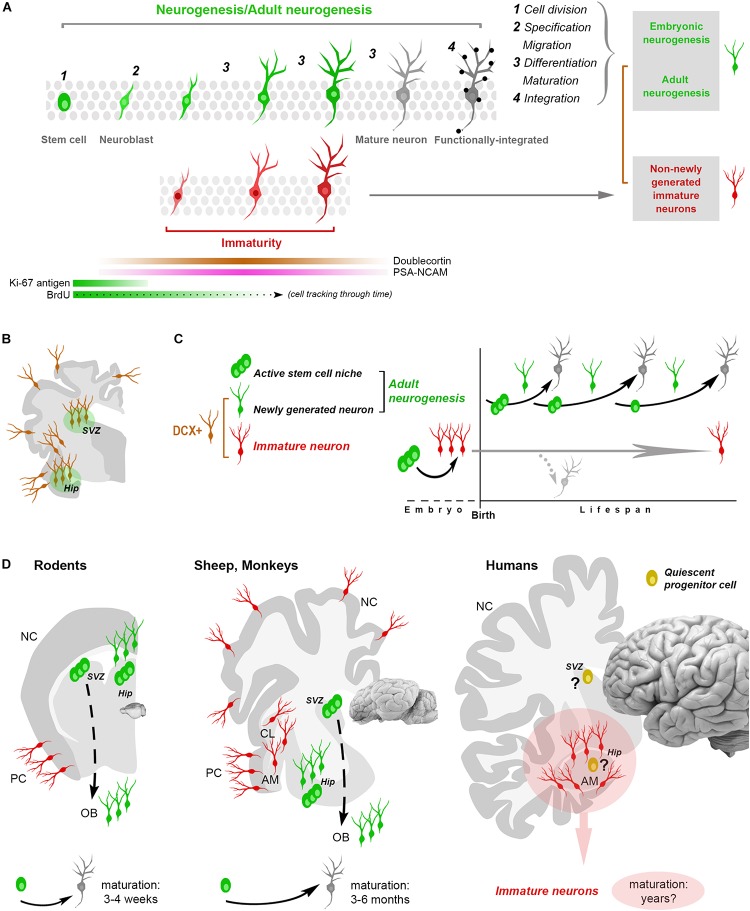
Shared aspects and differences in neurogenic and non-neurogenic processes. **(A)** Neurogenic events (both in embryo and adult) are multistep processes starting from stem cell division and coming out with the functional integration of mature neurons into the neural circuits. Immature neurons (INs; detectable with molecular markers of “immaturity” transiently expressed during the maturation process) represent only a phase in such a process. Gray rectangles on the right: different situations/developmental stages sharing a phase of neuronal immaturity. Color code: *green*, stem/progenitor cells, proliferative events and newly generated neurons; *red*, state of immaturity (shared by newly generated and non-newly generated neurons); *dark gray*, maturity (black dots, synaptic contacts); *brown*, doublecortin-immunoreactive (DCX+) cells. **(B,C)** The occurrence of DCX in the adult mammalian brain is no more an unequivocal proof that cells are newly generated since DCX is also expressed by populations of (non-newly generated) INs located in different brain regions (cerebral cortex, amygdala, claustrum and white matter, **B**). **(C)** At least two categories of DCX+ cells have been identified: newly generated (continuously produced within active neural stem cell niches) and non-newly generated INs. **(D)** Non-newly generated INs prevail in some large-brained, gyrencephalic mammals, which tend to show lower rates of adult neurogenesis and longer times of maturation for the newly generated neurons, what might explain the finding of many INs associated with a few proliferative events in the human hippocampus (*pink area:* current gap of knowledge). AM, amygdala; CL, claustrum; NC, neocortex; PC, paleocortex; OB, olfactory bulb.

The birth of neurons from NSC/radial glia cells has been well demonstrated both in embryonic and AN (Lim and Alvarez-Buylla, 2014; Berg et al., 2019). The germinal layers in the embryo and the neurogenic sites in the adult brain (subventricular zone, V-SVZ; subgranular zone, SGZ; hypothalamus) are microenvironments in which the NSCs are regulated so that new neurons can be formed. Hence, an adult neurogenic process, as we now understand it, must be sustained by an active NSC niche (Figure 1A). If we accept this definition, then the biological limits of mammalian AN are clear: it is highly restricted to small neurogenic zones, most cells proliferating outside these regions are glial cells, it is related to physiological needs and species-specific adaptations/behaviors, and it is strictly linked to the different animal species, developmental stages and ages (Bonfanti, 2016; Paredes et al., 2016).

Also, in the case of well-established NSC niches (V-SVZ and SGZ), the mainstream view that considers AN at the same level of other stem cell-derived regenerative processes is misleading. Even in mice, the rate of neurogenesis drops exponentially during life due to stem cell depletion (Ben Abdallah et al., 2010; Encinas et al., 2011; Smith et al., 2019), a condition that is very different from adult cell renewal processes in the body, which proceed at a steady rate throughout life (Semënov, 2019). The cells produced by hippocampal AN are not destined to fully and continuously replace old granular cells (as in blood or epidermis), but rather to provide a supply of new elements to complete the functional development of the dentate gyrus (Semënov, 2019). Whether quiescent progenitors can provide slow genesis of new neurons outside the neurogenic sites and in the absence of a niche remains to be demonstrated (Feliciano et al., 2015).

### Markers

The issue of detecting (and interpreting) structural plasticity in different mammalian brains is complicated by a substantial lack of highly specific markers. Biological events involving developmental stages (i.e., embryonic and AN) are dynamic, multistep processes characterized by transient gradients of molecular expression (Figures 1A,B). Most cellular markers available for this kind of research are necessarily ill-defined, since they are associated with developmental/maturational stages of the cells (dynamic changes of molecular gradients) that are not exactly the same in different cell populations, brain regions and/or animal species. For instance, markers of stem cells (Sox2, nestin) or newborn neurons (DCX, PSA-NCAM) are abundant in these cell categories but not exclusively associated with them, being detectable also in other contexts. The cytoskeletal protein DCX is also abundant in cells that are born prenatally, and then remain undifferentiated for long times by continuing to express immaturity molecules (INs, Gómez-Climent et al., 2008; Bonfanti and Nacher, 2012; König et al., 2016; Piumatti et al., 2018; Rotheneichner et al., 2018; Figures 1B–D). Considering DCX as a proxy for AN (as nestin was in the past for NSCs) or PSA-NCAM and DCX as markers for cell migration, are among the most common biases. A population of these cells, called cortical immature neurons (cINs), is resident in layer II of the adult cerebral cortex: the cINs are neither newborn nor migrating cells, though they heavily express DCX and co-express PSA-NCAM (Bonfanti and Nacher, 2012).

Before 2008, these features of “retained immaturity” where not known and we ignored that INs can also be found in extra-cortical regions (Luzzati et al., 2009; Bonfanti and Nacher, 2012; König et al., 2016; Piumatti et al., 2018). At that time, it was common to read statements like “DCX could be developed into a suitable marker for AN and may provide an alternative to BrdU labeling” (Brown et al., 2003), which is now questionable. The picture has changed and “time” has emerged as an important variable: the duration of “transient” marker expression in the cells, making more difficult to interpret cell maturation. The highly variable periods necessary for cell maturation/integration of neurons in different contexts (see below), along with their different origins (pre- or postnatal), introduce new nuances and further difficulties in determining which kind of plasticity is actually involved in different species, ages, and brain regions.

### Outcome

The final outcome of neurogenic processes (not intended as the phenotypic fate of the cells, but their survival over time) can be heterogeneous concerning both the single cells and the whole process. Apart from V-SVZ and SGZ, in which the ultimate functional integration into the olfactory bulb and hippocampus is well established, for other potential sources of new neurons the destiny of the progeny is far from clear. A third neurogenic site in the hypothalamus hosts an NSC-like niche that produces neurons with unclear fate, in terms of their final integration (Bonfanti and Peretto, 2011). Similarly, in ectopic examples of “parenchymal” neurogenesis (e.g., rabbit striatum and cerebellum; reviewed in Feliciano et al., 2015) the genesis of new neurons seems to be followed by their disappearance, suggesting a transient existence (Gould et al., 2001; Luzzati et al., 2014).

By considering the whole neurogenic process across time, its rate is progressively reduced with age, and the reduction is greater and faster in large-brained mammals (Paredes et al., 2016; Parolisi et al., 2018). Hence, a different outcome of AN can depend on the animal species. More generally, structural plasticity could be viewed as a progressive postnatal maturation of single brain regions/cell populations differing by location and time course, aimed at providing dynamic modulation based on life experiences. According to this view, AN in large-brained mammals would fall in the general rule of critical periods: temporal windows in which it is allowed, followed by the complete development of neural circuits (Semënov, 2019). It has been shown recently that mouse cINs can mature and be integrated into circuits at different ages (Benedetti et al., 2019), likely achieving a sort of “delayed neurogenesis.” A recent report showing an abundance of INs in the sheep brain (Piumatti et al., 2018) supports the hypothesis that these cells might represent an evolutionary choice in large-brained mammals, as an alternative/parallel form of plasticity (Palazzo et al., 2018).

By putting together origin, markers and timing of the maturation of different types of young neurons existing in the adult brain, the differences/similarities between AN and INs come into light: some markers are shared (DCX, PSA-NCAM), whereas the time of their expression and the origin of the cells (prenatal or postnatal) can be quite different (Figures 1A,B).

## Current State of the Art: Adult Neurogenesis or Immature Neurons for the Human Brain?

After some reports described a dramatic postnatal drop of neurogenesis in the human brain, occurring in the V-SVZ around the second year of life (Sanai et al., 2011) and in the hippocampal SGZ between age 5 and 13 years (Cipriani et al., 2018; Sorrells et al., 2018), other studies reported that neurogenesis was maintained in the human hippocampus (Boldrini et al., 2018; Moreno-Jimenéz et al., 2019; Tobin et al., 2019). However, in these latter studies, expression of molecular markers associated with stages of neuronal maturation (nestin, Sox2, DCX, and PSA-NCAM), was found mainly in large, ramified cells resembling INs, rather than the small, bipolar morphology typical of recently generated neuroblasts. Virtually all the studies (supporting or refuting existence of AN) failed to identify substantial rates of cell proliferation or a recognizable niche-like histological structure.

**FIGURE 2:**
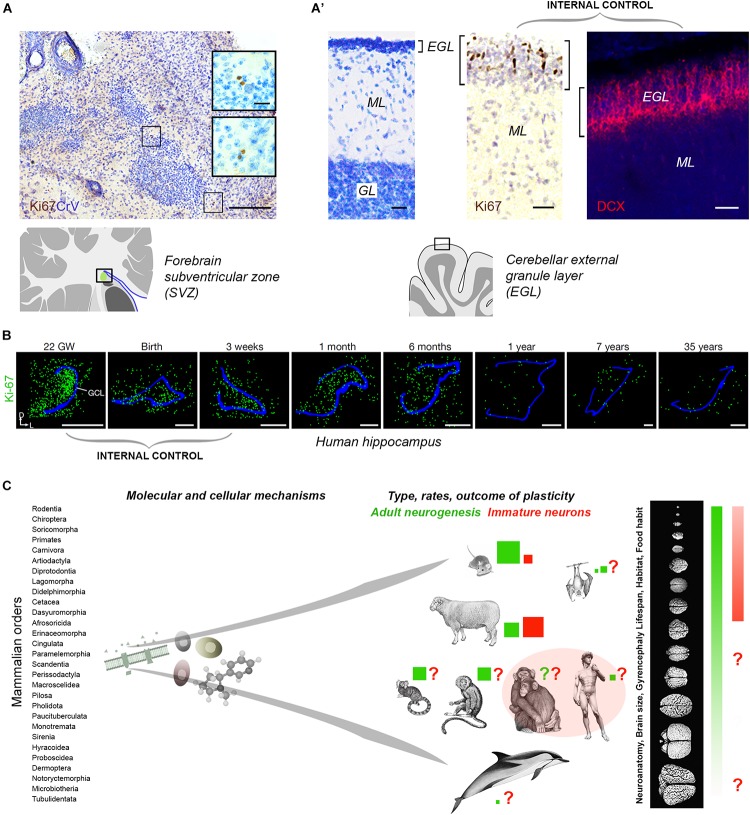
**(A,B)** Internal controls are needed for confirming the occurrence/absence of low/absent neurogenesis. Since most neurogenic processes substantially decrease with age, the detection of their markers at different time points (especially those related with cell proliferation), from early pre-postnatal stages to adulthood/aging, provides proof for their detectability in a given tissue. **(A)** Detection of very low rates of cell division (Ki-67 antigen) in the SVZ-like region of the neonatal dolphin, indicating that the periventricular germinal layer is already vestigial at birth. By contrast, a still highly proliferative external granule layer (EGL) is detectable in the cerebellum of the same animals **(A’)**. **(B)** Dramatic reduction of cell proliferation (green) in the dentate gyrus of the human hippocampus at different pre-, post-natal, and adult ages. Modified from Parolisi et al. (2017) **(A**,**A’)** and Sorrells et al. (2018) **(B)**; reproduced with permission from Springer Nature. **(C)** Beside common features shared at the cellular and molecular level, some complex biological processes, such as brain plasticity, can remarkably differ as a consequence of evolutionary differences among mammalian species. Left, mammals consist of around 30 orders of animals including more than 5.000 species highly differing for anatomy, physiology, behavior, habitat; right, the heterogeneity affects distinct neuroanatomy, brain size and computational capacities. Color code: red and green coherent with Figure 1; red and green square sizes indicate the importance of different types of plasticity in different species on the basis of the current literature (approximate estimation in the absence of systematic, comparable studies); *pink area*, current gap of knowledge concerning primates.

Tissue quality in non-perfused specimens (postmortem interval and fixation) is certainly important in detecting some markers: more DCX+ neurons were detected in human brain hippocampus by Moreno-Jimenéz et al. (2019) with respect to Sorrells et al. (2018). Yet, in non-perfused tissues, an internal positive control is required (Figures 2A,B). Sorrells et al. (2018) performed a complete histologic analysis using whole sections of hippocampus examined through pre-, postnatal and adult ages, thus providing an internal control for cell marker expression and its progressive drop over time (Figure 2B). In contexts providing the above mentioned internal controls, Ki-67 antigen staining for cell proliferation did work well in brain tissues extracted 18–40 h prior fixation, and then left in formalin for years (Parolisi et al., 2017; Figures 2A,A’). Aside from the number of cells detected, the DCX+ elements described in this way, without substantial proliferative activity, typical neuroblast morphology, or histological demonstration of a stem cell niche, cannot be considered an indication of “AN,” but rather of putative INs.

The origin and identity of the DCX+ cells in the human hippocampus remains to be determined: they look like young neurons in the absence of a proliferative niche, though located within a previously active neurogenic site. Something similar has been described in the human amygdala, wherein robust neurogenesis in the perinatal period is followed by an early drop of cell proliferation and persistence of DCX+ cells (Sorrells et al., 2019). This discrepancy is the current gap of knowledge: no sharp limits seem have been discovered between AN and INs in the human brain. On the basis of the currently available technical tools it is quite difficult to establish if some quiescent/slowly proliferating progenitors can be the source of these DCX+ neurons (also because similar processes are lacking in rodents). Reports in mammals living longer than mice indicate that the cells generated in their hippocampi mature across longer time courses (3 months in sheep, 6 months in monkeys, with respect to 3–4 weeks in rodents; Kornack and Rakic, 1999; Kohler et al., 2011; Brus et al., 2013; Figure 1D), thus suggesting that a slow, delayed maturation of neurons might replace neurogenic processes at certain ages. This hypothesis is coherent with the “preference” of INs in the relatively large sheep brain (Piumatti et al., 2018) and points to the possibility of a “reservoir of young neurons” in the mature brain of large-brained species (Palazzo et al., 2018; Rotheneichner et al., 2018; La Rosa et al., 2019).

## Current Research Gaps and Future Directions

Despite a huge amount of data on brain structural plasticity, many gaps of knowledge still remain unresolved, mainly concerning differences between rodents and humans, and the identity of the “young” neurons. We lack highly specific markers and the experience to interpret them in some contexts (e.g., the capability to discriminate among different types of plasticity involving different degrees of immaturity). We lack systematic and comparable studies encompassing very different animal species or different developmental stages/brain regions within a single species, carried out with standard protocols for fixation, tissue processing and cell counting methods. Particularly in humans, there is an urgent need to reproduce and confirm results. To fill these gaps, experimental approaches/tools are needed to study cell proliferation/survival processes that are slow and scattered (in space and time) in large brains.

## Key Concepts

Clarifying which types of plasticity can persist in the adult human brain is important for obvious translational purposes. Mice and humans share striking biological similarities, mainly regarding basic molecular mechanisms, yet important differences also emerge when complex biological processes are concerned (Figure 2C). There are substantial differences in the rate of AN and existence of INs among mammals: we are starting to learn that evolution might have sculpted multifaceted nuances instead of sharply defined processes. Since working directly on the human brain implies obvious ethical and technical limits, large-brained animal models are required. Dominant models may bias research directions or omit important context (Bolker, 2017); on the other hand, large animals are not easy to handle, and working on them is ethically disputable, time consuming and costly. The solution might consist of a mix of purposes, including: (i) rigorous adherence to the definition of AN to distinguish it from INs; (ii) development of new markers for better assessment of different phases of neuronal maturation; (iii) understanding of phylogenetic/evolutionary aspects of structural plasticity and their ramifications/adaptations in mammals; (iv) awareness that AN “function” remains substantially unsolved and that AN may not be a function, but rather a “tool” that the brains uses to perform/improve different functions based on different adaptations. Hence, the functions revealed in rodents can be specific to their ecological niche/behavior/needs (Amrein, 2015), and not fully transferable to humans. We must remember that there are no ends in science but only new, unexpected twists in the road driven by new technologies.

## Author Contributions

LB wrote the manuscript. CL and RP contributed to write the manuscript and performed the experiments allowing this mini-review to be written.

## Conflict of Interest

The authors declare that the research was conducted in the absence of any commercial or financial relationships that could be construed as a potential conflict of interest.
